# How to Improve the Antioxidant Defense in Asphyxiated Newborns—Lessons from Animal Models

**DOI:** 10.3390/antiox9090898

**Published:** 2020-09-21

**Authors:** Hanna Kletkiewicz, Maciej Klimiuk, Alina Woźniak, Celestyna Mila-Kierzenkowska, Karol Dokladny, Justyna Rogalska

**Affiliations:** 1 Department of Animal Physiology and Neurobiology, Faculty of Biological and Veterinary Sciences, Nicolaus Copernicus University, 87-100 Torun, Poland; kletkiewicz@umk.pl (H.K.); 259848@stud.umk.pl (M.K.); 2 Department of Medical Biology and Biochemistry, Faculty of Medicine, Collegium Medicum in Bydgoszcz, Nicolaus Copernicus University, 87-100 Torun, Poland; al1103@cm.umk.pl (A.W.); celestyna_mila@cm.umk.pl (C.M.-K.); 3 Department of Internal Medicine, School of Medicine, University of New Mexico, Albuquerque, NM 87131, USA; KDokladny@salud.unm.edu

**Keywords:** neonatal hypoxia/ischemia, therapeutic hypothermia, deferoxamine, oxidative stress, antioxidants, neuroprotection

## Abstract

Oxygen free radicals have been implicated in brain damage after neonatal asphyxia. In the early phase of asphyxia/reoxygenation, changes in antioxidant enzyme activity play a pivotal role in switching on and off the cascade of events that can kill the neurons. Hypoxia/ischemia (H/I) forces the brain to activate endogenous mechanisms (e.g., antioxidant enzymes) to compensate for the lost or broken neural circuits. It is important to evaluate therapies to enhance the self-protective capacity of the brain. In animal models, decreased body temperature during neonatal asphyxia has been shown to increase cerebral antioxidant capacity. However, in preterm or severely asphyxiated newborns this therapy, rather than beneficial seems to be harmful. Thus, seeking new therapeutic approaches to prevent anoxia-induced complications is crucial. Pharmacotherapy with deferoxamine (DFO) is commonly recognized as a beneficial regimen for H/I insult. DFO, via iron chelation, reduces oxidative stress. It also assures an optimal antioxidant protection minimizing depletion of the antioxidant enzymes as well as low molecular antioxidants. In the present review, some aspects of recently acquired insight into the therapeutic effects of hypothermia and DFO in promoting neuronal survival after H/I are discussed.

## 1. Introduction

Neurons have endogenous cellular systems to counteract neuronal damage [1]. Some of them prevent cell death and others allow functional recovery after injury. Once the neural network has been damaged, reconstruction to the state prior to the insult seems to be challenging. Thus, improving the effectiveness of this natural protection might help the remaining neural circuits to compensate for lost or broken circuits and enhance overall network performance and neurological function.

The pathophysiology of central nervous system damage due to neonatal H/I is complex. Recent research clearly suggests that free oxygen radicals are a key neurotoxic factor in that process [2,3]. Both enzymatic and non-enzymatic mechanisms of reactive oxygen species (ROS) scavenging are well-known players in neuron homeostasis. Harmful stimuli can lead to malfunctioning in one or more antioxidant defense systems affecting global redox balance and finally contributing to pathological conditions. Mobilization of the antioxidant system, including antioxidant enzymes and low-molecular antioxidants, is a substantial component of the protection against oxidative lesions.

H/I-induced brain lesions have a progressive nature. Their occurrence is not limited to H/I episode, but paradoxically their appearance accelerates when the delivery of oxygen-rich blood to the brain is restored. Delayed onset of serious lesions during oxygen restoration (reoxygenation) can appear in new-borns or even in adulthood and can manifest in a wide spectrum of neurodegenerative disorders. Sudden oxygen restoration leading to the overproduction of oxygen radicals following H/I seems to be the main pathological factor leading to delayed brain injury manifested by brain lesions [4]. 

H/I-induced brain lesions are closely associated with high levels of iron that is a cofactor in free-radical reactions. First, the content of free iron in the brain of neonates is much higher than in adults, as it is important for the proper maturation of the nervous tissue at the early stages of development [5]. Secondly, after brain H/I episode, iron deposited within neurons and released from microglia initiates a subsequent phase of free radical production [6,7,8,9]. Additionally, low levels of antioxidant enzymes (superoxide dismutase and glutathione peroxidase) and low-molecular antioxidants in new-borns make them particularly vulnerable to free radicals [10]. This means that changes in oxidative status during early life can interfere with normal brain development.

Body temperature maintained during brain H/I and reperfusion/reoxygenation is a very important factor affecting the extent of brain lesions. Decreased temperature greatly minimizes the intensity of harmful neuron-damaging reactions [11,12]. Reduction in body temperature by lowering the metabolism exerts a protective effect after cerebral ischemia allowing the diminished oxygen supply under asphyxic conditions to match its demand [13,14,15]. In animal models, decreased body temperature during neonatal asphyxia has been proven to reduce oxidative stress and to increase cerebral antioxidant capacity [16]. 

Clinical interest in therapeutic hypothermia (TH) began in the 1950s with case reports of successful resuscitation after immersing asphyxiated infants in a tub of cold water [17,18]. In the subsequent studies, a correlation between newborn infant’s body temperature and sequent mortality suggests that infant’s body temperature is not only a marker of neonate’s status but can also stimulate the development of lesions (fever or hyperthermia) or can be neuroprotective (hypothermia) [19]. In randomized clinical trials, therapeutic hypothermia has been shown to be the only treatment which improves outcomes in late preterm and full-term infants suffering from an encephalopathy of a hypoxic-ischemic origin [20]. 

In preterm or severely asphyxiated new-borns, therapeutic hypothermia, rather than beneficial, could be harmful [20]. Thus, it is essential to seek other clinically proven therapies to prevent H/I-induced complications. In numerous animal models, including mice [21], rats [22,23], and lambs [24], deferoxamine has been shown to alleviate H/I-mediated brain injury. DFO acts by chelating iron and alleviating oxidative stress. Recent studies have also shown that DFO initiates neuroprotective mechanisms by enhancing antioxidantdefense [25,26,27]. 

Animal models have been useful in replicating multiple pathophysiological features of neonatal H/I in humans. This research has helped to develop novel therapeutic targets and therapies for treatment of neonatal H/I [28]. In this review we focus on therapeutic hypothermia and DFO treatment in enhancing endogenous neuroprotective processes involved in the regulation of redox status under neonatal H/I conditions.

## 2. Animal Models of Neonatal Hypoxia/Ischemia

Over the past decades, a number of animal models, including both primate and sub-primate species, have been developed to mimic the pathophysiology of perinatal H/I-induced brain injury and its consequences. The brain development in newborn piglets shows similarities to that of human fetuses of 36–38 weeks of age [29]. In rabbits, similarly to humans, the maturation of oligodendrocytes begins antenatally and myelin formation occurs postnatally [30]. The stage of neurodevelopment of the preterm sheep fetus is similar to that of the 24- to 28-week human, whereas the late-gestation ovine fetus nervous system is comparable to that in full-term humans [31]. 

Only a few studies have investigated perinatal asphyxia (PA) in large animals such as pigs, sheep, or lambs [32,33,34], or in primates [35]. Three main procedures to develop H/I brain injury in piglets have been described: the combination of hypoxia and hypotension-induced ischemia [36], cardiac arrest followed by cardiopulmonary resuscitation [37], and bilateral common carotid artery occlusion combined with hypoxia [38]. More research has been performed in fetal sheep. In this model, the occlusion of the common uterine artery of pregnant sheep for 30–60 min, alone or in combination with supplementary maternal hypoxia for 120 min, has been previously carried out [39,40,41,42]. Another commonly used H/I model in fetal sheep involves ligation of vertebral-carotid arterial anastomoses and placement of occludes on both common carotid arteries [43,44]. PA in non-human primates is mostly employed to mimic profound asphyxia in the full-term neonate [45]. In rhesus monkeys, total asphyxia during delivery, produced by covering the heads and clamping the umbilical cords, resulted in neuropathological injury [35,46,47]. In other experiments, the induction of hypotension in pregnant monkeys resulted in partial asphyxia in a fetus [35]. Since the extent of asphyxia-induced brain injury in primates is similar to humans, they are regarded as an ideal animal model of human H/I [48]. Summarizing, large animals have been successfully used to replicate H/I-induced brain injury in humans. However, the main disadvantage of these models is lack of transgenic organisms or validated behavioral tests that are readily available in rodents. Moreover, ethical issues, advanced intensive care requirements, and high experimental costs are obvious restrictions that prevent the use of these models on a large scale [28]. Therefore, although larger animals resemble human physiology more closely, rodents are mostly used in neonatal H/I research [28]. In rats, there are several crucial stages in the brain development that occur in early postnatal period [49]. At postnatal days 1–3 (P1–3), the rat brain is comparable to the brain of immature preterm infants in age of 23–32 gestational weeks. The rat brain at P7 is similar to the brain at gestational stage of the human fetus at 32–34 week or of the preterm infant [50]. Thus, rodent H/I models replicating the clinical symptoms of H/I in neonatal humans are commonly used to investigate H/I-induced brain damage and in developing therapeutic strategies.

The most widely used animal model of neonatal HI is the Rice–Vannucci model (RVM) [51]. In this model, the carotid artery is unilaterally occluded followed by a period of hypoxic exposure (8% oxygen for 1–3 h at 37 °C) at P7. Although originally developed in rats [51], it has also been successfully adapted in other rodents at all stages of brain development [52,53,54,55,56]. This kind of damage recapitulates focal middle cerebral artery stroke-like pathology which is uncommon in human preterm infants [57,58]. The main disadvantage of the model is the necessity of anesthesia during surgical intervention.

Global asphyxia is another experimental model to induce the central nervous system damage that is associated with subsequent behavioral abnormalities. Asphyxia is induced by immersing fetus-containing uterus horns in a water bath at 37 °C for various time periods (0–22 min) [59,60]. Then, the pups are removed from the uterine horns and resuscitated. One significant drawback of this model is a high mortality rate when the water bath lasts more than 19 min [28,41,61]. 

Alternativemodels of neonatal asphyxia involving oxygen deprivation without ischemia have also been developed. These noninvasive models without the confounding effects of surgical procedures replicate milder injuries that are typical in asphyxiated human newborns. Lack of standardized procedures regarding age of the animals, duration of the insult, and body temperature seem to be challenging [62,63,64,65]. It should be emphasized that body temperature of newborn rats during the first days after delivery is 32–33 °C [66,67] and an increase in body temperature of newborn rats to 37 °C observed in adult rats should be referred to as hyperthermia. Hyperthermia of this degree increases the sensitivity of the immature rodent brain to hypoxia or ischemia intensifying brain damage [68]. Research procedures on animal models of neonatal H/I should consider thermoregulatory processes and age of the animals. Although cooling therapy has been used clinically in infants with hypoxic–ischemic encephalopathy, a search for the optimaltherapeutic hypothermia(TH) protocol is still ongoing.

## 3. Hypoxia/Ischemia-Induced Changes of Oxidative Status in the Brain

Neonatal H/I is a condition characterized by reduced oxygen levels and is the most common cause of death and disability in human new-borns [69]. Post-hypoxic injury occurs mainly in the areas of the human brain with a high metabolic rate and with a large number of excitatory glutamatergic neuronal synapses [70]. One of the major mechanisms leading to H/I-induced brain damage is excitotoxicity of glutamate neurotransmission leading to overactivation of postsynaptic receptors and cell death [69]. The other contributing factors to neonatal H/I injury are inflammation and oxidative stress [3]. 

**Figure 1:**
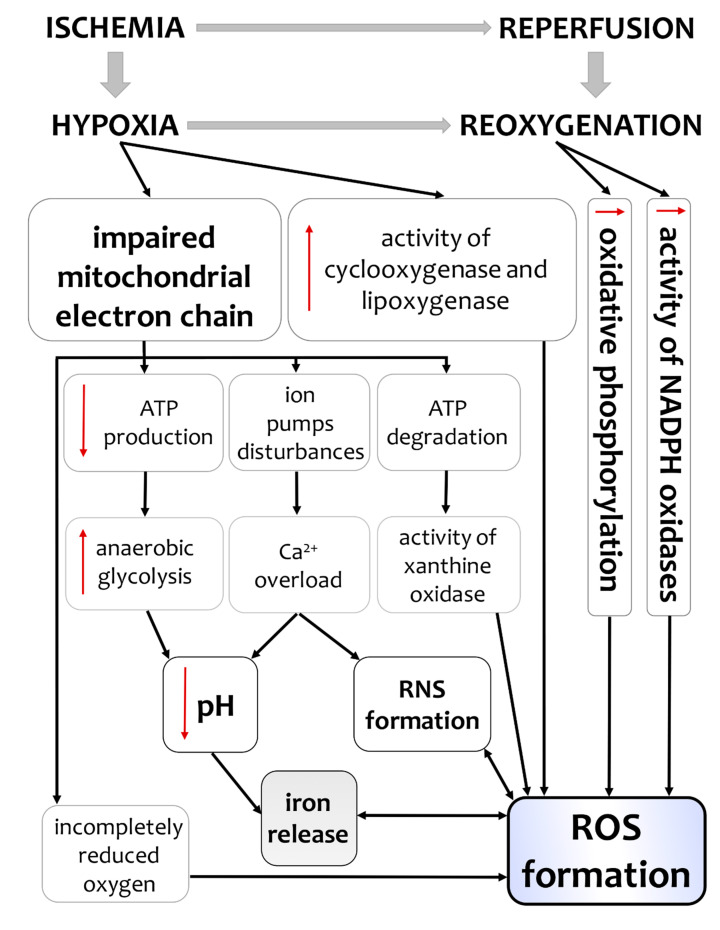
Mechanisms of ROS and RNS formation after hypoxia/ischemia (H/I) insult followed by reoxygenation/reperfusion. Abbreviations: ATP—adenosine triphosphate, NADPH—reduced nicotinamide adenine dinucleotide phosphate, ROS—reactive oxygen species, RNS—reactive nitrogen species.

An increase of reactive oxygen species (ROS) production after H/I followed by reoxygenation is well-established (Figure 1) [4]. Under hypoxic conditions, the main source of ROS is the mitochondrial electron transport chain. Low oxygen levels decrease its activity, thus promoting a certain amount of oxygen to be incompletely reduced cumulating in ROS overproduction, e.g., superoxide and hydroxyl radicals [71,72]. During reoxygenation, mitochondrial oxidative phosphorylation is overwhelmed leading to further accumulation of active oxygen species in hypoxic cells [73]. During H/I and reoxygenation, additional endogenous production of superoxide radicals also arises from reduced nicotinamide adenine dinucleotide phosphate(NADPH) oxidases (NOX) which are solely responsible for ROS formation [74,75]. 

An increased activity of xanthine oxidase also contributes to the augmented ROS formation following H/I. During such an insult, oxidative phosphorylation is impaired resulting in an increased adenosine triphosphate (ATP) degradation and accumulation of hypoxanthine [76]. During reoxygenation, hypoxanthine is metabolized by xanthine oxidase with concomitant production of superoxide radical and hydrogen peroxide [77]. 

In postischemic tissues, the activity of enzymes involved in the inflammatory process, including cyclooxygenase and lipoxygenase can also increase the ROS production [78]. During H/I, the enhanced ROS formation along with low pH (due to increased anaerobic metabolism, leading the elevated levels of lactic acid) are accompanied by an increased iron release from protein complexes [8,9]. Numerous studies have reported iron liberation from heme and ferritin during neonatal asphyxia [23,79,80,81]. In the presence of hydrogen peroxide (H_2_O_2_), iron can catalyze the formation of highly toxic hydroxyl radical (OH·) in a process called the Fenton’s reaction: Fe^2+^ + H_2_O_2_ = Fe^3+^ + OH + OH^−^ [82]. 

Under hypoxic conditions, not only ROS but also reactive nitrogen species (RNS) are overproduced. In cells subjected to H/I, disruption of ATP generation causes inhibition of ion pumps in the cell membrane and thus intracellular calcium overload [72,83]. Calcium is also responsible for nitric oxide synthase-induced nitric oxide (NO) formation. NO is the most detrimental RNS, which can significantly enhance the toxicity of superoxide radicals, including the synthesis of the most powerful oxidant-peroxy-nitrite (ONOO^−^) [84]. 

Mitochondria significantly contribute to ROS formation following H/I [85]. In neurons, mitochondria are a primary source of ROS formation and simultaneously the main target of excessively generated ROS [2]. ROS generated in mitochondria play a significant role in releasing cytochrome c and other pro-apoptotic proteins which can trigger caspase activation and apoptosis [86]. Disruption of mitochondrial function also leads to ATP depletion and necrotic cell death [87]. 

In response to an increased generation of ROS and RNS constituting a potential threat to the structure and function of the cells, activation of antioxidant defense mechanisms take place. The antioxidant enzymatic defense barrier is composed of three main enzymes: superoxide dismutase (SOD), catalase (CAT), and glutathione peroxidase (GPx) [72,88]. SOD catalyses the dismutation of superoxide radical to hydrogen peroxide, which in turn undergoes reduction with the participation of GPx and CAT [2]. There are three known superoxide dismutases: Cu, Zn-SOD (SOD1), Mn-SOD (SOD2) and the extracellular SOD-EC-SOD (SOD3). The presence of Cu, Zn-SOD was discovered in the cytoplasm, lysosomes, and in the mitochondrial intermembrane space [89], whereas Mn-SOD was found in the mitochondrial matrix [2]. In the brain, EC-SODprotect against ROS generated by membrane-bound NAD(P)H oxidase [90]. Vitamin E is a lipid-soluble scavenger that protects the brain against excessive lipid oxidation [2]. Other components of the defense mechanism include non-enzymatic endogenous (melatonin, thioredoxin, and glutathione) and exogenous (vitamin C, carotenoid, minerals, and polyphenols, including flavonoids) ROS scavengers [91]. 

Antioxidant enzymes are the most potent ROS scavengers protecting against cell damage under physiological conditions [92]. However, under asphyxic conditions the antioxidant barrier seems insufficient [93]. Following neonatal asphyxia, the disruption of oxidant/antioxidant balance of the oxidation processes leads to oxidative stress resulting in neuronal damage. This was proven both in animal models [26,27,65] and in babies suffering from birth asphyxia [81,94]. 

The developing brain is particularly prone to damage caused by ROS and RNS. This is related to several factors, including high demand of the brain for oxygen; high concentration of easily oxidizing polyunsaturated fatty acids in the brain tissue which constitute the main ingredient of phospholipids with low antioxidant defense; high concentration of metals catalysing ROS generation; and a large proportion of sensitive immature cells [95]. High ROS levels damage lipids (peroxidation of lipids), proteins (protein denaturation), and DNA initiating the cascade leading to cell death [96]. Nevertheless, ROS-induced protein oxidative modifications, such as the reversible oxidation of protein cysteine residues can play some beneficial roles [97]. Yan [98] postulates that redox modification of certain proteins, when induced purposely by approaches that trigger positive (weak) oxidative stress, can serve as a cellular defense mechanism that protects against ischemic injury.

## 4. Therapeutic Hypothermia—Impact on Oxidative Homeostasis under Hypoxic/IschemicConditions

Finding an effective treatment for asphyxiated infants has been a challenging task. Despite many experimental designs potentially effective in alleviating H/I insult, hypothermia remains the only clinically proven treatment for newborns [20,99]. Research shows that hypothermia protects against brain damage after asphyxiation by reducing oxidative stress [11,12,16]. 

Horiguchi et al. [100] demonstrated that post-ischemic hypothermia completely inhibited hydroxyl radical and xanthine formation in the striatum in rats. Mild hypothermia also reduced superoxide anion generation in the penumbra and the corresponding contralateral region following focal cerebral ischemia in rats [14]. Moreover, Huang et al. [101] reported that hypothermia significantly reduced the levels of ROS and NO in the mouse brain after H/I. Decreased expression of nitric oxide synthase (iNOS) was also observed as a result of hypothermia combined with administration of N-acetylcysteine in neonatal rats after severe H/I injury [102]. Cerebral metabolic rate decreases by approximately 6–7% for every 1 °C drop in body temperature, thereby diminishing oxygen demand and decreasing ROS formation [103]. 

As previously stated, H/I followed by reoxygenation significantly increases the production of reactive oxygen species [4]. ROS are responsible for the release of iron from binding proteins, which is then deposited in the brain causing a cascade of neurotoxic events leading to brain injury [80]. Reduced body temperature was proven to diminish anoxia-induced release of iron, as well as its accumulation in the brain of rats [23,104], thuspreventing secondary free radical formation.

One of the most commonly investigated consequences of ROS activity within cellular structure and function is lipid peroxidation. Isoprostanes are now regarded as the gold standard of oxidative stress assessmentin vivo [105]. Huun et al. [106] found that hypothermia following H/I in new-born piglets reduces the isoprostane compound 8-iso-PGF_2α_levels in urine, but none of the other lipid peroxidation compounds were affected by the hypothermia treatment. Research on the H/I model in new-born piglets has shown that hypothermic conditions caused the main effect in the white matter, where it significantly reduced lipid peroxidation products such as F4-neuroprostanes, F2-isoprostanes, and DihomoIsoprostanes [107]. The other commonly used lipid marker of oxidative stress is malondialdehyde (MDA) [108]. Kletkiewicz et al. [27] have shown that hyperthermic conditions (37 °C and 39 °C) increase MDA concentration provoking post-anoxic oxidative stress. However, in rats with body temperature kept at 33 °C, no increase in anoxia-induced MDA level was observed. On the contrary, Toader et al. [109] have found that a single hypothermia episode increased MDA levels compared to controls. However, MDA levels were significantly lower in the group subjected to H/I insult with prior hypothermia exposure.

In cells, reactive oxygen species may promote deleterious effects by protein carbonylation, which is a type of protein oxidation [110]. It has been demonstrated that post-asphyxic hypothermia significantly reduces protein carbonyl formation in the parietal cortex and the striatum in newborn piglets 6h after acute H/I [23,111]. However, no significant changes were observed in protein carbonyl levels in the brain tissue when comparing hypothermic and normothermic piglets 48h after H/I insult [112]. The authors have postulated that oxidative stress plays an important role in brain damage in the first hours after H/I, returning progressively to basal levels thereafter. On the other hand, H/I followed by 29 h of hypothermia increased the level of carbonylated proteins as compared to normothermia in new-born piglets. Similar results were observed in the white matter of the brain in neonatal piglets exposed to asphyxic cardiac arrest followed by short-duration hypothermia (6 and 20 h) [113]. DNA repair gene expression (DNA glycosylases OGG1, NEIL1 and NEIL3) was reduced in hypothermic pigs compared to the control or resuscitated group [114]. The decreased levels were mostly evident in the hippocampus, but also in the cortex, cerebellum, and liver.

Although hypothermia treatment is considered the gold standard after H/I insult, in some new-borns and infants adverse outcomes have been reported [111]. Recent research focuses on the development of therapies that can be used in combination with hypothermia to enhance its neuroprotective effects. Significantly reduced contents of MDA, ROS, and NO after combined crocin and hypothermia treatment, rather than hypothermia alone, were found in the mouse brain after H/I [101]. The combination of cannabidiol and hypothermia led to decreased protein carbonyl levels in H/I-treated piglets. This may suggest that the combined therapy provides some additive effects leading to more complete neuroprotection than cannabidiol or hypothermia alone [111,112]. Moreover, early treatment with the inhibitor of 20-hydroxyeicosatetraenoic acid (20-HETE), a compound which contributes to oxidative stress, might offer a strategy to enhance the clinical efficacy of delayed hypothermia after H/I insults in the developing brain [115]. 

Antioxidant defense in the developing brain is less active. Research to seek factors which will not only decrease ROS generation but will also increase antioxidant properties is of significant importance [116,117]. 

According to Kletkiewicz et al. [26], body temperature influences the concentration of low-molecular antioxidants in the asphyxiated brain of newborn rats. In neonates kept at hyperthermic conditions (body temperature of 37 °C and 39 °C), cerebral concentration of vitamin E and the reduced form of glutathione (GSH) was lower than in rats kept at normothermic body temperature (i.e., 33 °C, the physiological body temperature of newborn rats). Moreover, in normothermic rats, the post-anoxic pool of low-molecular antioxidants were not depleted [26]. Vitamin E is the most important lipophilic radical scavenging antioxidant [118]. It belongs to the secondary antioxidants as it reacts with ROS that have already been formed causing their removal or inhibition [15]. GSH, on the other hand, is the main intracellular antioxidant in the CNS [119]. The more ROS is produced, the more GSH is used, leading to the decrease in its concentration [120]. In the cell, at least 90% of glutathione is present in a reduced form. Decreased GSH levels and increased levels of an oxidized form (glutathione disulfide—GSSG) indicate a distortion of oxidative-antioxidant balance towards oxidative stress [121]. The liver is a main organ for GSH production and release into plasma [122]. Alva et al. [123] indicated an increase in GSSG/GSH ratio in liver homogenates of adult rats subjected to hypoxia in normothermia. In a group of animals exposed to hypoxia and treated with hypothermia, this ratio was comparable to the control group, i.e., to animals exposed only to hypothermic conditions without hypoxia. Reduced glutathione is an active enzyme, which undergoes oxidation to GSSG in a reaction catalyzed by GPx [124]. In rats exposed to hypoxic conditions, both in normothermia and hypothermia, a lower concentration of GSH was observed in the liver. Unexpectedly, in the hypothermia-hypoxia group, a reduced concentration of GSSG was observed [123]. In human newborns with hypoxic-ischemic encephalopathy treated with hypothermia, an increase of GSH concentration in the thalamus and white matter was found. However, in the basal ganglia or cortical grey matter, no significant changes in these endogenous antioxidant levels were observed [125]. 

In new-born rats kept at their physiological body temperature of 33 °C, there was no post-anoxic decrease in SOD, GPx or CAT activity [27], whereassignificantly lower antioxidant enzyme activities in the brain occurred in anaerobic as well as normoxic new-born rats kept in hyperthermic (37 and 39 °C) and profound hypothermic (31 °C) conditions. The activities of these antioxidant enzymes were, however, lower in anoxic rats [126]. 

Postischemic mild hypothermia delayed the utilization of SOD, GPx and GSH in the brain tissue [127]. Hypothermia led to an increase in SOD-1 expression in the neocortex and in the caudate-putamen in a rat model of neonatal hypoxic–ischemic encephalopathy [128]. The increase in GPx-1 expression occurred in the caudate-putamen, but not in the neocortex.

Nuclear factor erythroid 2-related factor 2 (Nrf2) is a key molecule involved in maintaining redox balance [129]. Enhancement of MnSOD activity and expression via Nrf2 activation has been shown in mitochondria isolated from pig brains subjected to mild hypothermia [130]. In new-borns subjected to hypoxic and ischemic insult followed by hypothermia, a significant enhancement of antioxidant defense was observed manifested by an increased expression of SOD and GPx [109]. On the contrary, SOD activity in the ischemic core as the effect of lowered generation of superoxide radical anion was slightly reduced in hypothermia-treated rats compared with normothermic animals exposed to focal cerebral ischemia [14]. 

Total antioxidant status (TAS) of the blood was elevated in infants subjected to perinatal asphyxia followed by therapeutic hypothermia (33–34 °C for 72 h) in comparison with new-borns kept at normothermic conditions [131]. Therapeutic hypothermia (32–33 °C) applied after severe traumatic brain injury in infants and children (within either 6 h or 24 h following the injury, and for 48 h) resulted in increased glutathione levels in the cerebrospinal fluid (CSF). A similar reverse relationship was observed between temperature and total antioxidant reserve in CSF [132]. These results were not confirmed when total antioxidant properties in the serum were assessed by the biological antioxidant potential (BAP) of asphyxiated infants subjected to hypothermia or normothermia [15]. Oxidative stress index (calculated as the ratio of total hydroperoxide to BAP) was significantly lower in hypothermia than normothermia.

The impact of hypothermia on oxidative stress is not unambiguous.Some studies show no beneficial effect of therapeutic hypothermia, whereas others demonstrate an increase, rather than decrease, in oxidative stress under these conditions. Under hypothermic conditions, both a decrease in oxidative stress and a slight increase in mitochondrial superoxide anion radical production, as well as its decreased dismutation, were reported [109]. Hypothermia without any hypoxic insult has been shown to stimulate oxidative stress conditions in new-born rats [109]. Moreover, deep hypothermia (31 °C) intensifies post-anoxic oxidative stress and depletes the antioxidant pool, and thus may extend perinatal anoxia-induced brain lesions [27]. Table 1 presents the research directly verifying the impact of TH on oxidative status under H/I conditions.

## 5. DFO—A PromisingAgent in Hypoxic/Ischemic Encephalopathy Therapy?

In the treatment of perinatal asphyxia resulting in hypoxic–ischemic encephalopathy (HIE), therapeutic hypothermia is a current standard of care. Nevertheless, the efficacy of TH in some clinical cases is still unclear [1,111,133,134]. Additionally, since its discovery, little progress has been made in identifying new pharmacological therapies.

We will discuss potentially successful pharmacological intervention with DFO which seems to be a promising agent against hypoxic-ischemic brain injury. DFO, also referred to as desferoxamine, desferrioxamine, or by its brand name Desferal (DFO), is the most widely investigated iron-chelator for brain injury treatment. In recent years, several mechanisms describing the efficacy of DFO have been postulated.

Generally, iron chelators are used as antioxidants. The antioxidant activity of DFO was first attributed to its iron binding capacity [135]. Under physiological conditions, iron is stored in proteins such as ferritin or hemosiderin and therefore does not induce free radical production. Following perinatal asphyxia, iron could be released from the binding proteins [80,136]. Moreover, after asphyxia the blood pH decreases, causing transferrin to release iron. As a result, free iron can induce the production of free radicals and can also accumulate in the brain causing progressive degeneration of the nervous tissue [137]. Ferrous (Fe^2+^) ions may stimulate the generation of malondialdehyde (MDA), a lipid peroxidation product recognized as an indicator of oxidative tissue injury [138]. Additionally, these ions leak from plasma through the damaged blood–brain barrier into the brain and are absorbed directly by brain cells. DFO, after treatment, rapidly passes the blood–brain barrier [24]. Through binding ferric irons, DFO prevents the formation of the highly damaging peroxynitrite anion as well as hydroxyl radicals via the Fenton/Haber–Weiss reaction [24,139,140]. Consequently, DFO decreases the potential for iron-mediated oxidative free radical damage, most significantly polyunsaturated lipids peroxidation. The efficacy of DFO application in the alleviation of lipid peroxidation level after hypoxic-ischemic brain injury was proven in new-born piglets [141]. Similarly, a DFO-mediated reduction in brain iron content and decrease of lipid peroxidation after H/I was shown in lambs [24]. In 7-day-old rats, DFO treatment reduced the risk of iron-dependent injury [22]. The beneficial effect of DFO administered in 2-day-old rats after anoxia event has also been observed. Post-anoxic injection of DFO prevented iron accumulation in the frontal cortex, hippocampus and striatum three weeks after anoxic event [23]. The reduction of iron content and neuroprotective effect of DFO in a mouse model of cerebral H/I have also been shown [21]. DFO not only sequesters “free iron”, but also reduces hydroxyl radical generation and cell death in both P7 hippocampal cultures exposed to oxygen–glucose deprivation (OGD) and in the brains of neonatal rats exposed to H/I [142]. 

The second mechanism underlying the neuroprotective action of DFO is free radical scavenging. It was shown that DFO acts as a chain-breaking radical scavenger, donating an electron or hydrogen atom from the hydroxamate centre [143]. In hippocampal neuronal cultures pre-treatment with DFO reduced the damage produced by H_2_O_2_. Moreover, pre-treatment with DFO reduced cell death in neurons exposed to a sublethal concentration of H_2_O_2_ [25]. 

Free radicals impair transmembrane Na^+^/K^+^-ATPase activity by inducing depolarization of the cell membrane and releasing a large amount of glutamic acid. There is strong evidence that the release of neurotransmitters such as glutamate, which stimulates α-amino-3-hydroxyl-5-methyl-4-isoxazole-propionate (AMPA) and N-methyl-D-aspartate (NMDA) receptors, leads to excitotoxicity of cells and generation of free radicals [144,145,146,147]. In large animal models (pigs and lambs) with H/I injury, besides reducing free iron levels, DFO preserves cerebral energy metabolism and electrical brain activity, as well as attenuating the brain edema [24,148,149]. It may also protect the injured immature brain by decreasing glutamate and aspartate levels, which are increased under asphyxic conditions causing excitotoxicity [150]. Moreover, DFO pre-treatment protects immature primary hippocampal neurons against NMDA (glutamate agonist)-induced toxicity [25]. 

Under hypoxic conditions, DFO also induces hypoxia-inducible factor-1 alpha (HIF-1α), which plays a fundamental role in the regulation of oxygen homeostasis [151]. The impact of ROS on HIF-1α production is somewhat controversial. Some studies have proposed that ROS production decreases HIF-1α accumulation probably by restoring prolyl hydroxylase (PHD) activity, the enzyme responsible for HIF-1α degradation [152,153]. In this context, iron chelators prevent ROS production by promoting HIF-1α stabilization. These findings have been further supported by the results in in vitro studies showing that DFO pre-treatment significantly reduced production of ROS in an oxygen and glucose deprivation (OGD) model [154]. Additionally, DFO has been shown to increase the expression of HIF-1α target genes, including vascular endothelial growth factor (VEGF) and erythropoietin (EPO) [155,156]. VEGF is a polypeptide growth factor which promotes cell survival by stimulating angiogenesis [157]. EPO, on the other hand, is a humoral mediator involved in erythropoiesis and provides significant neuroprotection during neonatal hypoxia [158,159]. It was also proven that blocking HIF-1α reduces DFO’s neuroprotective effect suggesting that DFO may protect the neural tissue through HIF-1α induction [160]. In vivo studies have also shown that DFO can up-regulate HIF-1α expression in both a rat neonatal stroke model [161] and a neonatal H/I model [162]. 

Less characterized but notable actions of DFO include suppression of anoxia/hyperthermia (39°C)-induced antioxidant enzymes, low molecular weight antioxidantdepletion, and prevention of lipid peroxidation [26,27]. The neuroprotective role of DFO under anoxic/hyperthermic conditions was also supported in behavioral experiments. The neonatal rats exposed to hyperthermia alone and those exposed to anoxia at elevated body temperatures showed disturbances in behavioral responses and spatial memory impairment throughout their lives, while post-anoxic DFO treatment prevented such disturbances [23,49]. Table 2 presents the studies concerning the neuroprotective role of DFO, promoting cell survival under H/I conditions.

## 6. Conclusions

Current knowledge allows us to conclude that endogenous neuroprotectants can determine the extent of hypoxia/ischemia induced brain injury. The brain mounts a defensive response against H/I-induced brain injury; however, in case of serious damage, this is only partially successful. From a therapeutic perspective, it is crucial to develop therapies that enhance the neuroprotective mechanisms or alleviate the pathologies under H/I conditions. An antioxidant system seems to be the most promising endogenous target for experimental and translational therapy. As free radicals are involved in many pathways leading to cell death, by modulating oxidative status it is possible to affect whole cell survival potential. Increasing evidence has indicated that intentionally induced oxidative stress can exert great beneficial effects on cellular adaptation to stress challenges and cell survival. In this context, protein redox modifications may be used as a potential therapeutic target for attenuating tissue ischemic injury, in particular reversible modifications of protein cysteine residues induced as preconditioning or postconditioning.

There are many identified candidate substances and factors potentially therapeutic for nervous system rescue after asphyxic insult. It is challenging to identify that with the most neuroprotective value. The best effects can be achieved when the therapy inhibits more than one pathological pathway. It seems that therapeutic hypothermia matches these criteria. Therapeutic hypothermia represents the factor that regulates the majority of intracellular processes, important in rescuing cell homeostasis, including antioxidant status. Research presented in this review has shown that body temperature during hypoxia/ischemia and/or reoxygenation/reperfusion affects oxidative status in the brain. Simulated neonatal hypoxia/ischemia in animals treated with decreased body temperature alleviates oxidative stress and increases the levels of the antioxidant enzymes, as well as low-molecular antioxidants. Successful therapy should be focused on level of body temperature decrease because excessive cooling might counteract the potential benefit of a moderate decrease in body temperature.

If therapeutic hypothermia is not effective, especially in preterm or seriously asphyxiated new-borns, other therapies should be considered.

Existing data on mechanisms underlying the neuroprotective action of DFO imply that DFO acts not only via iron chelation, but also by enhancing antioxidative status. Its role in improving antioxidant defense under H/I conditions (both alone or combined with hypothermia) requires further investigation before being translated into clinical practice. In order to ensure optimal and long-term protection, protocol optimalization of DFO usage, including timing and ideal dosing considering the level of injury, age, gender of new-born, and comorbidities is urgently needed. It is also possible that combined therapies consisting of therapeutic hypothermia and a pharmacological agent might be more effective than a single regimen for H/I injury. Progress in this area of research could benefit from integrated analyses combining knowledge gained from cell culture studies, as well as animal and human research. We can also expect that with new technological developments novel therapies may arise. One of the most promising is single-cell RNA sequencing (scRNA-seq), which for example can reveal complex and rare cell populations, uncover regulatory relationships between genes, and track the trajectories of distinct cell lineages in development. Thus, these innovations should be included into research on animal models. We hope that ongoing and future studies investigating neuroprotectants and their mechanisms will develop novel therapies for H/I-induced disorders.

## Figures and Tables

**Table 1:** Effects of therapeutic hypothermia on oxidative stress.

References	Animal Models	Sample Size	Hypoxic/Anoxic Damage	Temperature	Impact on Oxidative Stress
Barata et al., 2019	male piglets; one-day-old	No data	Oxygen decreased to 10% for 25 min.	Normothermia: 37.5–38.5 °C (rectal) Hypothermia: 34–34.5 °C (rectal)	↑ oxidative stress after H/I; ↓ protein carbonyl levels (markers of oxidative stress) after the combination of cannabidiol and hypothermia
Dalen et al., 2010	Noroc (LYxLD) pigs; newborn (25 ± 4.8 h of age)	52, weight: 1980 ± 106 g,	Oxygen decreased to 8% for 61 min.	Normothermia: 39 °C (rectal) Hypothermia: 35 °C (rectal)	↑ oxidative stress after hypoxia; ↓ expression of genes of DNA repair after hypothermia, with no effect on accumulation of oxidative damage in genomic DNA
Huang et al., 2019	C57BL/6J mice; post-natal day 7	no data	Oxygen decreased to 8% for 30 min	Normothermia: 36 °C (rectal) Hypothermia: 33 °C (rectal)	↓ ROS and NO as a result of hypothermia ↓ MDA, ROS and NO after hypothermia combined with crocin
Huun et al., 2018a	Noroc (LyxLD) pigs; newborn (12–36 h of age)	55	Oxygen decreased to 8% for 20 min	Normothermia: 38.5–39.5 °C (rectal) Hypothermia: 34.5 °C (rectal)	↓ oxidative stress markers: 8-iso-PGF_2α_ (in urine) after hypoxia and hypothermia
Huun et al., 2018b	Noroc (LyxLD) pigs; newborn (12–36 h of age)	81	Oxygen decreased to 8% for 20 min	Normothermia: 39 °C (rectal) Hypothermia: 34.5 °C (rectal)	↓ oxidative stress markers: F_4_-NeuroPs, F_2_-IsoPs, DH-isoprostanes (in the white matter) after hypoxia and hypothermia
Kletkiewicz et al., 2016a	Wistar rats; two-days-old	108, both sexes, weight: 7–8 g	100% nitrogen atmosphere for 10 min	Normothermia for newborn rat: 33 °C (rectal) Hyperthermia: 37–39 °C (rectal)	↑ lipid peroxidation ↓ antioxidant enzymes activity after perinatal anoxia at elevated body temperatures, however there was no decrease in enzymes activity in the group with body temperature of 33°C
Kletkiewicz et al., 2016b	Wistar rats; two-days-old	180, both sexes, weight: 7–8 g	100% nitrogen atmosphere for 10 min	Normothermia for newborn rat: 33 °C (rectal) Hyperthermia: 39 °C (rectal)	Normothermic (33°C) body temperature prevents post-asphyxic disturbances in cerebral oxidant homeostasis (markers: level of low-molecular antioxidants)
Kletkiewicz et al., 2016c	Wistar rats; two-days-old	192, both sexes, weight: 7–8 g	100% nitrogen atmosphere for 10 min	Hypothermia: 31 °C (rectal) Normothermia for newborn rat: 33 °C (rectal) Hyperthermia: 37 °C & 39 °C (rectal)	↑ MDA, ↑ CD and ↓GPx in both hyper-thermic groups ↑ SOD and ↓ CAT in extremely hypothermic and hyperthermic newborns, no changes in the levels of° MDA, CD and in enzymes activity in rats with body temperature of 33 °C
Lafuente et al., 2016	male piglets; 1 to 2-day-old	no data	Oxygen decreased to 10% for 30 min	Normothermia: 38 °C (rectal) Hypothermia: 33–34 °C (rectal)	↓ protein carbonyls formation in parietal cortex and striatum 6h after H/I and hypothermia, cannabidiol enhance the protective effect of hypothermia
Mueller-Burke et al., 2008	male piglets; 5 to 7-day-old	26, weighing 3.0–4.5 kg,	Oxygen decreased to 10% for 30 min	Normothermia: 38.5 °C (rectal) Hypothermia: 34 °C (rectal)	↓ protein oxidation after post-hypoxic mild whole-body hypothermia
Nie et al., 2016	Sprague-Dawley rats; post-natal day 7	21	Oxygen decreased to 8% for 120 min	Normothermia: 36.3 ± 0.5 °C Hypothermia: 30 ± 0.5 °C	↓expression of nitric oxide synthase (iNOS) after post-hypoxic hypothermia combined with N-acetylcysteine
Santos et al., 2018	male piglets; 2 to 3-day-old	98, weight: 1.0–2.5 kg	Oxygen decreased to 10% for 45 min	Normothermia: 38.0 to 39.5 °C (rectal), Hypothermia: 34.0 °C	↑ carbonylated protein levels after H/I and hypothermia
Toader et al., 2013	Wistar rats; Newbornpost-natal day 7	80, both genders, weight: 10 g	Oxygen decreased to 8% for 90 min	Normothermia: no data on value Hypothermia: 33–34 °C (intra-rectal),	↑ MDA ↓ SOD and GPx in hypothermia, ↓ MDA ↑ SOD in H/I and hypothermia
Zhu et al., 2014	male piglets; 3–5 days of age	50	Oxygen decreased to 10% for 45 min	Normothermia: 38.5 to 39 °C (rectal), Hypothermia: 34.0 °C	the use of inhibitor of oxidative stress promoter enhances the effect of delayed hypothermia

Abbreviations: H/I—hypoxia-ischemia, ROS—reactive oxygen species, GSH—reduced glutathione, NO—nitric oxide, MDA—malondialdehyde, SOD—superoxide dismutase, CAT—catalase, GPx—glutathione peroxidase.In order to prepare the table, bibliography research in PubMed was performed using the following keywords in varied combinations: “hypoxia”, “ischemia”, “asphyxia “, “anoxia”, “hypothermia”, “temperature”, “oxidative stress”. We also used Boolean operator “and” to receive the most relevant search results. All articles written in English were manually screened, and the appropriate articles were identified.Only those published from 2000 to 2020 were taken into consideration. Papers concerning the effects of hypothermia on brain oxidative status under neonatal hypoxia/ischemia/asphyxia or anoxia, evaluated in animal models, were considered.

**Table 2:** Effects of deferoxamine (DFO)on brain oxidative status and other markers of brain injury.

References	In Vitro/In Vivo Models	DFO Dose/Time of Administration	Hypoxic/Ischemic Damage	Suggested Mechanisms of Action	Result of DFO Administration
Papazisis et al., 2008	Wistar rats; seven-day-old	150 mg/kg s.c; subcutaneously, immediately after insult and 24 h later	Oxygen decreased to 8% for 60 min	impact on the neurotransmitters’ release	decreases the excitatory amino acid levels; reduces the number of damaged neurons in the CA1 region
Kletkiewicz et al., 2016a	Wistar rats; two-days-old	100 mg/kg s.c; subcutaneously, immediately after insult and 24 h later	100% nitrogen atmosphere for 10 min	antioxidant action	prevents SOD, CAT and GPx depletion; decreases MDA level
Kletkiewicz et al., 2016b	Wistar rats; two-days-old	100 mg/kg s.c; subcutaneously, immediately after insult and 24h later	100% nitrogen atmosphere for 10 min	antioxidant action	prevents cerebral glutathione and vitamin E depletion; decreases MDA level
Chu et al., 2010	Primary astrocyte cultures from PD1–2 Swiss white mice	Preconditioning for 0.5 to 24 h with 0.1–1 mM of DFO	hydrogen peroxide exposure (0.1–1 mM) for a further 24 h	iron chelation - removing the PHD-bound ferrous ion	protects the astrocytes against H_2_O_2_-induced injury; changes the expression of HIF-1α and VEGF
Hamrick et al., 2005	Hippocampal neurons from E16 CD1 mice	pretreatment with 10 mmol/L DFO for 1h	95% N and 5% CO_2_ for 5 min	iron chelation	reduces cell death; induces HIF-1α
Almli et al., 2001	Primary hippocampal cell culture from fetal (E16) CD-1 mice	Pretreatment for 1 h at various doses ranging 50–20 mM	H_2_O_2_ and NMDA exposure for 24 h	antioxidant action and iron chelation	reduces cell death; protects against H_2_O_2_ and NMDA-induced toxicity
Sarco et al., 2000	tg mice, carrying the SOD1 gene; seven-day-old	100 mg/kg s.c; subcutaneously, immediately after insult and 24h later	Oxygen decreased to 8% for 30 min	iron chelation	reduces brain iron content and hypoxic-ischemic brain damage
Feng et al., 2000	Piglets; 0 to 3-days-old	100 mg/kg s.c; 15 min after recovery	Oxygen decreased to 6% for 15 min	iron chelation	inhibits lipid peroxidation
Peeters-Scholte et al., 2003	Piglets; 1 to 3-days-old	10 mg/kg upon reperfusion and a repeated dose of 2.5 mg/kg at 12 h, injected intravenously	1 h of hypoxia-ischemia by occluding both carotid arteries and reducing the fraction of inspired oxygen	iron chelation	maintains cerebral energy status after global hypoxia-ischemia
Lu et al., 2015	P7 hippocampal slice cultures exposed to oxygen–glucose deprivation (OGD)	100 mM; 2 h before OGD	Slices exposed to 0.1% O_2_, 5% CO_2_, 94.4% nitrogen for 90 min	iron chelation	reduces hydroxyl radical levels and neuronal cell death
Mu et al., 2005	Sprague–Dawley rats; ten-days-old	200 mg/kg s.c.; immediately after reperfusion administered intraperitoneally	middle cerebral artery (MCA) occlusion	Induction of HIF1α expression	increases HIF-1α and EPO level
Rogalska et al., 2006	Wistar rats; two-days-old	100 mg/kg s.c; subcutaneously, immediately after insult and 24 h later	100% nitrogen atmosphere for 10 min	iron chelation	protects against the brain hyperferremia
Caputa et al., 2005	Wistar rats; two-days-old	100 mg/kg s.c; subcutaneously, immediately after insult and 24 h later	100% nitrogen atmosphere for 10 min	iron chelation	prevents the behavioral disturbances

Abbreviations: GSH—reduced glutathione, MDA—malondialdehyde, SOD—superoxide dismutase, CAT—catalase, GPx—glutathione peroxidase, PHD—prolyl hydroxylase, H_2_O_2_—hydrogen peroxide, HIF-1α—hypoxia-inducible factor-1 alpha, VEGF—vascular endothelial growth factor, NMDA—N-methyl-D-aspartate, EPO—erythropoietin.In order to prepare the table, bibliography research in PubMed was performed using the following keywords in varied combinations: “neonatal”, “hypoxia”,“ischemia”, “anoxia”, “hypothermia”, “antioxidant”, “oxidative stress”, “deferoxamine”, “iron”. We also used Boolean operator “and” to receive the most relevant search results. All articles written in English were manually screened, and the appropriate articles were identified.Only those published from 2000 to 2020 were taken into consideration. Articles on the effects of deferoxamine on brain oxidative status and other markers of brain injury under neonatal hypoxia/ischemia/asphyxia or anoxia, evaluated in in vitro/in vivo models, were included.
